# Supplementary material for: Baby Foods: 9 Out of 62 Exceed the Reference Limits for Acrylamide
Source: Foods. 2024 Aug 26;13(17):2690. doi: 10.3390/foods13172690 (PMC11394281; doi:10.3390/foods13172690)
Supplement: Supplementary file 1 [file foods-13-02690-s001.zip › Table S1- REV1.pdf]

Table S1. Description of 62 samples analyzed and their acrylamide concentration ± standard deviation. For each sample, two replicates (N=2) of the AA extraction were performed and analyzed individually in LC-MS.

Sample	Product	Ingredients	Average nutritional values per 100 g	Production technology	Acrylamide concentration (µg/Kg) ± standard deviation	Coefficient of variation
OFR1	Organic homogenized apple and pear with peach percoca	Apple puree (39,96%), pear puree (35%), peach puree (25%), and ascorbic acid	54 kcal, 0 g fat, 12 g carbohydrates, 7.9 g sugar, 0.5 g protein, 0.01 g salt	Sterilization	2.78 ± 0.17	6,12
OFR2	Organic homogenized banana and apple	Banana puree (65%), apple puree (33%), lemon juice, and ascorbic acid	83 kcal, 0 g fat, 18 g carbohydrates, 13 g sugar, 0.9 g protein, 0 g salt, 2.5 g fiber	Sterilization	n.d.	n.d.
OFR3	Organic homogenized mixed fruit	Apple (53%), banana (20%), apricot (10%), water, orange juice from concentrate (7%), rice starch, and ascorbic acid	58 kcal, 0 g fat, 13 g carbohydrates, 10 g sugar, 0 g protein, 0.05 g salt, 1.4 g fiber	Sterilization	n.d.	n.d.
OFR4	Homogenized mixed fruit	Fruit 90%: (apple puree and juice, apricot, banana, juice from orange concentrate), water, corn starch, lemon juice concentrate, and vitamin C	60 kcal, 0.3 g fat, 14 g carbohydrates, 9 g sugar, 0.4 g protein, 0.01 g salt, 50 mg Vit C	Sterilization	n.d.	n.d.
OFR5	Homogenized apple and apricot	Apple puree (64.98%), apricot puree (35%), vitamin C	45 kcal, 0 g fat, 10 g carbohydrates, 8.3 g sugar, 0.5 g protein, 0.01 g salt	Sterilization	n.d.	n.d.
OFR6	Homogenized four fruits	Fruit 99.8%: (apple puree and juice concentrate, banana puree, apricot puree,	70 kcal, 0.5 g fat, 16 g carbohydrates, 14.5 g sugar, 0.5 g protein,	Sterilization	n.d.	n.d.

		orange juice concentrate), lemon juice concentrate, and vitamin C	0.004 g salt, 50 mg Vit C			
OFR7	Homogenized plum	Plum (84%), water, corn starch, lemon juice concentrate, and vitamin C	68 kcal, 0.2 g fat, 16 g carbohydrates, 10.5 g sugar, 0.5 g protein, 0.004 g salt, 50 mg Vit C	Sterilization	4.77 ± 0.25	5,24
OFR8	Homogenized plum	Plum (99.9%) and vitamin C	78 kcal, 0.2 g fat, 17.5 g carbohydrates, 14 g sugar, 0.6 g protein, 0.01 g salt, 50 mg Vit C	Sterilization	9.27 ± 0.23	2,48
OFR9	Homogenized plum with apple	Plum (44%), water, apple puree (15%), rice flour, and vitamin C	74 kcal, 0 g fat, 17 g carbohydrates, 11 g sugar, 0.6 g protein, 0.01 g salt, 18 mg Vit C	Sterilization	5.99 ± 0.13	2,17
FRU1	100% pear puree with vitamin C	Pear puree (99.96%) and vitamin C	53 kcal, 0 g fat, 11 g carbohydrates, 11 g sugar, 0.5 g protein, 0.01 g salt, 3.7 g fiber	Sterilization	2.94 ± 0.28	9,52
FRU2	100% mixed fruit puree with vitamin C	Mixed fruit puree (99.96%): (apple, pear, banana, apricot), and vitamin C	58 kcal, 0 g fat, 13 g carbohydrates, 13 g sugar, 0.5 g protein, 0.01 g salt, 2 g fiber	Sterilization	2.94 ± 0.28	4,78
FRU3	Organic pear puree	Pear puree	64 kcal, 0.2 g fat, 14 g carbohydrates, 14 g sugar, 0.5 g protein, 0.01 g salt, 2 g fiber	Sterilization	3.35 ± 0.16	n.d.
FRU4	Organic golden apple puree	Apple puree (99.99%)	56 kcal, 0.5 g fat, 11 g carbohydrates, 9.6 g sugar, 0.5 g protein, 0.01 g salt	Sterilization	n.d.	n.d.

OVE1	Homogenized carrots, potatoes and zucchini	Vegetables 70%: (carrots 35%, potatoes 25%, zucchini 10%), cooking water	27 kcal, 0.2 g fat, 5 g carbohydrates, 1.5 g sugar, 0.6 g protein, 0.04 g salt	Sterilization	16.80 ± 0.57	3,39
OVE2	Organic homogenized of mixed vegetables	Vegetables in variable proportion 77%: (carrots, potatoes, zucchini), cooking water	34 kcal, 0.5 g fat, 5.5 g carbohydrates, 1.8 g sugar, 1.1 g protein, 0.05 g salt, 1.3 g fiber	Sterilization	n.d.	n.d.
OVE3	Organic homogenized of mixed vegetables	Vegetables 73%: (carrots 39%, potatoes 14%, cauliflower, peas 10%), cooking water, sunflower seed oil	40 kcal, 1.3 g fat, 4.7 g carbohydrates, 2.2 g sugar, 1.2 g protein, 0.05 g salt, 2.4 g fiber	Sterilization	2.60 ± 0.28	10,77
OVE4	Homogenized beans with carrots	Cooking water, borlotti beans (22%), carrots (8%), rice flour (3%), and lemon juice concentrate	43 kcal, 0.3 g fat, 6.6 g carbohydrates, 0.7 g sugar, 2.3 g protein, 0.03 g salt	Sterilization	4.60 ± 0.57	12,39
OVE5	Homogenized chickpeas	Cooking water (59%) and chickpeas (41%)	43 kcal, 0.9 g fat, 4.9 g carbohydrates, 0 g sugar, 2.5 g protein, 0.01 g salt	Sterilization	n.d.	n.d.
OFO1	Organic homogenized with cheese	Water, cheese (43%: quark, 30%; cheddar, 8%; parmesan, 5%), rice starch, and sodium citrate	115 kcal, 5.6 g fat, 8.4 g carbohydrates, 1 g sugar, 7.7 g protein, 0.48 g salt, 0 g fiber	Sterilization	8.89 ± 0.46	5,17
OFO2	Homogenized molten cheese	Water, cheese (40%: milk, milk enzymes, salt, and rennet), corn starch, sodium citrate, and potassium citrate	138 kcal, 7.5 g fat, 5.5 g carbohydrates, 0 g sugar, 12 g protein, 0.9 g salt, 400 mg Calcium	Sterilization	5.96 ± 0.18	3,02
OCP1	Homogenized with chicken meat and rice flour	Cooking water, chicken meat (30%), corn starch, rice flour (2%), rice starch,	71 kcal, 2.4 g fat, 6.3 g carbohydrates, 0 g sugar, 6.1	Sterilization	211.84 ± 16.53	7,80

		and sunflower oil	g protein, 0.08 g salt			
OCP2	Organic homogenized chicken	Cooking water, chicken meat (40%), rice starch, and lemon juice	73 kcal, 2.5 g fat, 4.2 g carbohydrates, 0 g sugar, 8.5 g protein, 0.09 g salt, 0 g fiber	Sterilization	154.32 ± 12.71	8,24
OCP3	Organic homogenized with chicken meat and rice starch	Cooking water, chicken meat (30%), and rice starch (8%)	82 kcal, 3.6 g fat, 6.8 g carbohydrates, 0 g sugar, 5.7 g protein, 0.08 g salt, 0 g fiber	Sterilization	194.88 ± 7.40	3,80
OCPR1	Homogenized with ham and cereal	Cooking water, ham (30%), rice flour (10%), and lemon juice concentrate	83 kcal, 2.9 g fat, 7.3 g carbohydrates, 0.1 g sugar, 6.8 g protein, 0.07 g salt	Sterilization	2.02 ± 0.14	6,93
OCPR2	Homogenized with ham and rice flour	Cooking water, ham (30%), corn starch, rice flour (2%), rice starch, sunflower oil, and lemon juice concentrate	77 kcal, 2.6 g fat, 7.1 g carbohydrates, 0 g sugar, 6 g protein, 0.06 g salt	Sterilization	2.57 ± 0.07	2,72
OCPR3	Homogenized with ham and cereal	Cooking water, ham (20%), corn starch, rice flour (3%), and lemon juice concentrate	66 kcal, 2.5 g fat, 6.5 g carbohydrates, 0.1 g sugar, 4.5 g protein, 0.03 g salt	Sterilization	n.d.	n.d.
OCPR4	Organic homogenized with cooked ham and rice starch	Cooking water, cooked ham (30%), and rice starch (8%)	75 kcal, 2.5 g fat, 6.4 g carbohydrates, 0 g sugar, 6.7 g protein, 0.38 g salt, 0 g fiber	Sterilization	n.d.	n.d.
OCV1	Homogenized with veal and cereal	Cooking water, veal (30%), rice flour (10%), and lemon juice concentrate	100 kcal, 4.8 g fat, 7.6 g carbohydrates, 0.1 g sugar, 6.7 g protein, 0.08 g salt	Sterilization	5.35 ± 0.21	3,93
OCV2	Homogenized with veal and cereal	Cooking water, veal (20%), cornstarch, rice flour (3%), and lemon juice concentrate	70 kcal, 3.1 g fat, 6.3 g carbohydrates, 0.1 g sugar, 4.3 g protein, 0.05 g salt	Sterilization	11.87 ± 0.29	2,44

OCV3	Organic homogenized with veal and rice starch	Cooking water, veal (30%), and rice starch (8%)	71 kcal, 2.2 g fat, 6.8 g carbohydrates, 0 g sugar, 5.9 g protein, 0.08 g salt, 0 g fiber	Sterilization	10.66 ± 0.01	0,09
OPE1	Homogenized with sea bream and vegetables	Cooking water, vegetables 23.5% (potatoes, onions, celeriac), sea bream fillet (18%), cornstarch, rice flour, sunflower oil, and lemon juice concentrate	70 kcal, 1.8 g fat, 9.4 g carbohydrates, 0 g sugar, 3.7 g protein, 0.03 g salt	Sterilization	3.56 ± 0.04	1,12
OPE2	Homogenized with sea bass and potatoes	Cooking water, sea bass fillet (20%), potatoes (18%), rice flour (8%), extra virgin olive oil (0.3%), and concentrated lemon juice	77 kcal, 2.5 g fat, 9.2 g carbohydrates, 0.1 g sugar, 4.3 g protein, 0.05 g salt	Sterilization	n.d.	n.d.
OPE3	Organic homogenized with plaice and potatoes	Cooking water, potatoes (20%), plaice fillet (18%), rice flour, onions (2%), and sunflower oil	59 kcal, 1.3 g fat, 8.4 g carbohydrates, 0 g sugar, 3.2 g protein, 0.05 g salt, 0 g fiber	Sterilization	45.52 ± 0.28	0,62
OPE4	Homogenized with flounder and vegetables	Cooking water, vegetables 21.5% (potatoes, onions, celeriac, carrots), flounder fillet (18%), corn starch, rice starch, sunflower oil, and lemon juice concentrate	57 kcal, 0.6 g fat, 10 g carbohydrates, 0 g sugar, 2.7 g protein, 0.08 g salt	Sterilization	4.94 ± 0.01	0,20
SE1	Organic wheat semolina	100% durum wheat semolina, vitamin B1	384 kcal, 1.2 g fat, 81 g carbohydrates, 2.6 g sugar, 11 g protein, 0.05 g salt, 3.8 g fiber	Grinding	46.07 ± 0.23	0,50

SE2	Wheat semolina	Durum wheat semolina and vitamin B1	376 kcal, 1.3 g fat, 77 g carbohydrates, 1.1 g sugar, 12 g protein, 0.01 g salt, 1.4 mg Vit B1	Grinding	26.82 ± 0.26	0,97
CR1	Organic cream of rice	Rice flour, vitamin B1	379 kcal, 0.7 g fat, 85 g carbohydrates, 0 g sugar, 7.3 g protein, 0.05 g salt, 1.4 g fiber, 1.3 mg Vit B1	Grinding	22.84 ± 0.88	3,85
CR2	Cream of rice	Rice flour, vitamin B1	387 kcal, 1.2 g fat, 86 g carbohydrates, 0.5 g sugar, 7.7 g protein, 0.02 g salt, 0.90mg Vit B1	Grinding	20.85 ± 1.36	6,52
BV1	Vegetable broth	Dehydrated vegetables 43.5%: (potatoes, green beans, zucchini, carrots, parsley), maltodextrin, cornstarch, yeast extract, natural flavors, and rice flour	370 kcal, 0.5 g fat, 80 g carbohydrates, 6.5 g sugar, 8 g protein, 0.4 g salt, 6.4 g fiber	Freeze-drying	2.77 ± 0.18	6,50
B1	Children's biscuit with milk cream	Wheat flour (63%), sugar, vegetable oils, skim milk powder, milk cream (2%), barley malt extract, leavening agents, calcium carbonate, and flavorings.	419 kcal, 9 g fat, 74.7 g carbohydrates, 22 g sugar, 8.5 g protein, 0.4 g salt, 2.6 g fiber, 350 mg Vit A, 6 mg Vit E	Drying	276.36 ± 0.03	0,01
B2	Children's biscuit with -30% sugar	Wheat flour (63%), sugar, vegetable oils, soluble fiber from corn, skim milk powder, malt extract	399 kcal, 8.5 g fat, 67 g carbohydrates, 16 g sugar, 9.4 g protein, 0.37 g salt, 8.4 g	Drying	242.06 ± 0.78	0,32

		from barley, wheat starch, leavening agents, calcium carbonate, and flavoring	fiber, 0.5 mg Vit B1			
B3	Organic soluble biscuits for infants	Type 0 wheat flour, brown sugar, diastased soft wheat flour, sunflower seed oil, rice flake flour, butter, skim milk powder, raising agents, and barley malt extract	419 kcal, 8.5 g fat, 76 g carbohydrates, 26 g sugar, 8.5 g protein, 0.25 g salt, 2.3 g fiber, 0.8 mg Vit B1	Drying	108.15 ± 6.73	6,22
B4	Childhood biscuit	Wheat flour (77%), sugar, sunflower seed oils, skim milk powder, raising agents, flavorings, and vitamin B1	419 kcal, 8 g fat, 77 g carbohydrates, 22 g sugar, 8.5 g protein, 0.18 g salt, 0.8 mg Vit B1	Drying	234.78 ± 4.53	1,93
B5	Biscuits 6 cereals for infants	Cereal flours (68%): (wheat, oats, barley, rye, rice, corn), sugar, palm oil, malt from barley, raising agents, flavoring agents, and vitamins	410 kcal, 7.5 g fat, 76 g carbohydrates, 24 g sugar, 8 g protein, 0.8 g salt, 2.7 g fiber, 0.5 mg Vit B1	Drying	40.74 ± 1.74	4,27
B6	Organic spelt baby biscuits, milk, and egg free.	Wheat flour (spelt) (54%), wheat flour type 0, cane sugar, sunflower seed oil, raising agents, and vitamin B1	454 kcal, 14 g fat, 73 g carbohydrates, 20 g sugar, 8 g protein, 0.18 g salt, 1.7 g fiber, 0.8 mg Vit B1	Drying	66.01 ± 1.68	2,55
SN1	Paff with pumpkin and carrot	Cornmeal (74%), sunflower oil, pumpkin powder (7%), carrot powder (7%): (carrot 60%, rice flour), thiamine	438 kcal, 12.8 g fat, 72 g carbohydrates, 4.1 g sugar, 7.3 g protein, 0.06 g salt, 2.9 g fiber, 1.3 mg Vit B1	Drying	3.72 ± 0.20	5,38

SN2	Organic veggie triangles	Potato flakes (61%), rice flour, vegetables (5%: spinach, carrot, and red beet), and turmeric	327 kcal, 1.1 g fat, 67 g carbohydrates, 2.3 g sugar, 8.5 g protein, 0.2 g salt, 7.3 g fiber	Drying	n.d.	n.d.
SN3	Organic corn rings	Corn flour	389 kcal, 0.9 g fat, 87 g carbohydrates, 0.5 g sugar, 7.6 g protein, 0.01 g salt, 1.4 g fiber	Drying	25.88 ± 0.21	0,81
ME1	Children's snack milk and cocoa	Milk (91%), sugar, tapioca starch, low-fat cocoa (1.2%), and thickeners	85 kcal, 3.1 g fat, 9.8 g carbohydrates, 9.2 g sugar, 3.4 g protein, 0.09 g salt, 100 mg Calcium	Sterilization	40.95 ± 0.32	0,78
ME2	Milk and cocoa snack	Rehydrated milk powder (68%), water, sugar, rice starch, rice flour, cocoa powder (2.2%), and flavorings	88 kcal, 3 g fat, 12 g carbohydrates, 9.8 g sugar, 3 g protein, 0.08 g salt, 78 mg Calcium	Sterilization	20.79 ± 0.82	3,94
LP1	Nutri1 Infant Milk Powder	Milk with 3.2% fat (66%), demineralized whey powder, vegetable oils, maltodextrin, lactose from milk, galacto-oligosaccharides from milk, and skim milk fermented with *Lactobacillus paracasei* (0.9%)	507 kcal, 26.7 g fat, 55 g carbohydrates, 40 g sugar, 10.5 g protein, 0.44 g salt, 3.5 g fiber, 39 g lactose	Spray-drying	2.28 ± 0.08	3,51
LP2	Nutribiotik infant milk powder	Lactose, vegetable oils, skim milk powder, demineralized whey, milk galacto-oligosaccharides, milk serum protein	482 kcal, 24.6 g fat, 53.5 g carbohydrates, 52.7 g sugar, 9.7 g protein, 4.2 g fiber, 50.8 g lactose, 53 mg inositol	Spray-drying	n.d.	n.d.

		concentrate, fructo-oligosaccharides, and fish oil				
LP3	Follow-on milk 2 powder	Lactose, vegetable oils, skim milk powder, demineralized whey, milk galacto-oligosaccharides, milk serum protein concentrate, fructo-oligosaccharides, calcium phosphate, and milk protein	468 kcal, 22 g fat, 56.3 g carbohydrates, 55.5 g sugar, 9.4 g protein, 53.7 g lactose, 52 mg inositol	Spray-drying	n.d.	n.d.
LP4	Follow-on milk 2 powder	Demineralized whey, maltodextrin, vegetable oils, skim milk, cream, mineral salts, fish oil, choline birtrate, and vitamins	511 kcal, 26.4 g fat, 57.8 g carbohydrates, 43 g sugar, 10.6 g protein, 0 g fiber, 42 g lactose, 114 mg inositol	Spray-drying	13.60 ± 0.13	0,96
LC1	Growth Milk Soya3	Water, maltodextrin, vegetable oils, soy protein isolate, glucose, and fructose	72 kcal, 3.5 g fat, 7.5 g carbohydrates, 3.1 g sugar, 2.5 g protein, 0.1 g salt, 0 g fiber	UHT	11.43 ± 0.45	3,94
LC2	Growth Milk 3	Skimmed milk, water, lactose, milk galacto-oligosaccharides (2.3%), vegetable oils, and emulsifier	66 kcal, 2.7 g fat, 8.6 g carbohydrates, 8.4 g sugar, 1.5 g protein, 0.07 g salt, 0.8 g fiber	UHT	7.98 ± 0.28	3,51
LC3	Growth Milk 3	Whole milk (54.6%), demineralized water, milk lactose, milk galacto-oligosaccharides, maltodextrin, sucrose, vegetable oils,	69 kcal, 2.9 g fat, 8.5 g carbohydrates, 7 g sugar, 1.9 g protein, 0.075 g salt, 0.8 g fiber	UHT	n.d.	n.d.

		and mineral salts				
LC4	Growth Milk	Partly skimmed milk (52.6%), water, lactose, maltodextrin, vegetable oils, vitamin A, and vitamin B1	62 kcal, 3 g fat, 7.1 g carbohydrates, 4.9 g sugar, 1.7 g protein, 0.07 g salt, 0 g fiber	UHT	n.d.	n.d.
LIO1	Freeze-dried homogenized veal	Veal meat (85%), rice flour, and cornstarch	For 10 g: 45 kcal, 1.3 g fat, 3.6 g carbohydrates, 0.01 g sugar, 4.6 g protein, 0.04 g salt	Freeze-drying	n.d.	n.d.
LIO2	Freeze-dried homogenized veal	Veal meat and freeze-dried rice flour	For 10 g: 43 kcal, 1.1 g fat, 3.8 g carbohydrates, 0.005 g sugar, 4.5 g protein, 0.04 g salt	Freeze-drying	15.19 ± 0.37	2,44
LIO3	Freeze-dried homogenized turkey	Turkey meat (85%), rice flour, and cornstarch	For 10 g: 44 kcal, 1.2 g fat, 3.3 g carbohydrates, 0.01 g sugar, 5 g protein, 0.05 g salt	Freeze-drying	21.22 ± 1.49	7,02
LIO4	Freeze-dried homogenized rabbit	Rabbit meat (85%), rice flour, corn starch, and vitamin E	For 10 g: 44 kcal, 1.2 g fat, 3 g carbohydrates, 0.01 g sugar, 5.4 g protein, 0.04 g salt, 1.4 mg Vit E	Freeze-drying	9.80 ± 0.35	3,57
