# Supplementary material for: Baby Foods: 9 Out of 62 Exceed the Reference Limits for Acrylamide
Source: Foods. 2024 Aug 26;13(17):2690. doi: 10.3390/foods13172690 (PMC11394281; doi:10.3390/foods13172690)
Supplement: Supplementary file 1 [file foods-13-02690-s001.zip › Table S2 - REV1.pdf]

Table S2. Body weight (kg) in male and female subjects according to age [15].

Age (months)	Weight (kg)		
	**Male**	**Female**	**Average value**
6	7.9	7.3	7.6
12	9.6	8.9	9.25
24	12.2	11.5	11.85
36	14.3	13.9	14.10
